# Numerical Modeling and Thermovision Camera Measurement of Blast Furnace Raceway Dynamics

**DOI:** 10.3390/ma18133061

**Published:** 2025-06-27

**Authors:** Sailesh Kesavan, Joakim Eck, Lars-Erik From, Maria Lundgren, Lena Sundqvist Öqvist, Martin Kjellberg

**Affiliations:** 1 Swerim AB, Box 812, 97125 Luleå, Sweden; sailesh.kesavan@swerim.se (S.K.); maria.lundgren@swerim.se (M.L.); 2 Konstruktionstjänst AB, Björkövägen 64, 97561 Luleå, Sweden; lars-erik.from@bahnhof.se; 3 Minerals and Metallurgical Engineering Department, Luleå University of Technology, 97187 Luleå, Sweden; lena.sundqvist-oqvist@ltu.se; 4 SSAB EMEA AB, SSAB Special Steels Division, Aspaleden 2, 61331 Oxelosund, Sweden; martin.kjellberg@ssab.com

**Keywords:** blast furnace, raceway, pulverized coal, coal combustion, injection, CO_2_ emission, modeling, thermovision camera

## Abstract

The blast furnace (BF) and basic oxygen route account for approximately 70% of the global steel production and create 1.8 tons of CO_2_ per ton of steel, produced primarily due to the use of coke and pulverized coal (PC) at the BF. With global pressure to reduce CO_2_ emissions, optimization of BF operation is crucial, which is possible through optimizing fuel consumption, and improving process stability. Understanding the complex combustion and flow dynamics in the raceway region is essential for enhancing reducing agent utilization. Modeling plays a key role in predicting these behaviors and providing insights into the process; however, validation of these models is crucial for their reliability but difficult in the complex and hostile BF raceway region. In this study, a validated raceway model developed at Swerim was used to evaluate four different cases, namely R1 (Reference), R2 (Low oxygen to blast), R3 (High blast moisture), and R4 (High PC) using an injection coal from SSAB Oxelösund. During actual experiments, the temperature distribution in the raceway was measured using a thermovision camera (TVC) to validate the CFD simulation results. The combined use aims to cross-validate the results simultaneously to establish a reliable framework for future parametric studies of raceway behavior under varying operational conditions using CFD simulations The results indicated that it is possible to measure the temperature within the raceway region using TVC at depths indicated to be 0.5–0.7 m, when not obscured by the coal plume, or <0.5 m, when obscured. TVC measurements are clearly quantitatively affected when obscured, indicated by considerably lower temperatures in the order of 200 °C between similar process conditions. A decrease of O_2_ injection results in an extended raceway region as the conditions become less chemically favorable for combustion due to a lower reactant content offsetting the ignition point and reducing the reaction rate in the raceway. An increased moisture content in the blast results in a reduced size of the race-way region as energy is consumed as latent energy and cracks water. An increase in PC rate results in a larger/wider raceway region, as more PC is devolatilized and combusted early on, resulting in larger gas volumes expanding the raceway region outwards, perpendicular to the injection.

## 1. Introduction

Although efforts are being made toward greener steelmaking routes, the blast furnace (BF) route remains the dominant production method for steel in Europe, contributing to about 7% of CO_2_ emissions. Reducing greenhouse gas emissions is a key focus for European steelmakers which can be achieved by partially replacing coke with pulverized coal injection (PCI) or by injection of biocoal. The overall need for fossil coal can be reduced, as each kg of coke produced requires 1.25–1.4 kg of coal.

High coke replacement at high PC injection rates (PCR) requires the optimization of gas distribution to achieve high gas efficiency, as the reducing gas generated at the raceway must be efficiently distributed and used in the indirect reduction of iron oxide. At high PCR, the theoretical flame temperature drops, which is counter measured by increased oxygen enrichment to blast, and the ore/coke ratio in the burden increases [1]. The maximum oxygen enrichment to the blast is mainly limited by the minimum top-gas temperature at which moisture in charged burden can be evaporated [2]. In addition, the flame temperature is also lowered more if a high volatile coal is used, the blast temperature lowered, or the blast moisture increased. Although industrial research has been conducted, requirements of stable BF operation make studies difficult on an industrial scale, and experiments on a laboratory scale [3] and pilot scale [4] are widely used instead. The harsh environment inside an industrial scale BF limits process monitoring opportunities to external monitoring; therefore, is the use of computational fluid dynamics (CFD) combined with available process data a comprehensive tool to increase knowledge?

Several 3D models take this into account through different approaches, such as tuyere combustion models [5,6], tuyere–raceway combustion models [7,8,9,10] and tuyere–raceway–coke bed combustion models [9,11,12,13]. Raceway combustion models can solve detail particle dispersion due to the rather small computational domain. However, this has an impact on particle dispersion [4], and the results cannot be applied directly for conclusions about the BF raceway combustion efficiency. In the tuyere–raceway models, the raceway cavity was represented by a simplified geometry, and this approach gives the opportunity to resolve the free shear flow (jet) in the raceway region, capturing its effect on particle dispersion and combustion efficiency. Interactions with coke were neglected and the chemical reactions and gas composition were based only on pulverized coal (PC) combustion and gasification. The tuyere–raceway–coke bed combustion models include combustion of coke. The raceway shape was not explicitly calculated but was defined as a boundary condition to limit computational efforts. The raceway was represented by a total void balloon-shaped cavity by Shen et al. [9,11,12], while Maier et al. [13] used a more circular shape with gradients in void fraction in the raceway boundary. Both were based on literature sources, but a difference in gas flow field in the raceway cavity was observed.

The model by Maier involved coke bed movement and showed that inter-phase momentum transfer made coke particles recirculate in the upper part of the raceway, fall into the jet, and partly combust. This behavior was reported from experimental observations with a high-speed camera [14] as well as within experiments conducted in the RFCS project IMPCO [15]. In addition, a 3D CFD model was developed at Swerim, following the tuyere–raceway modeling approach to ensure detailed particle trajectories in the blast. In addition to similar tuyere–raceway models, coke interaction and subsequent chemical reactions were included to properly evaluate the combustion in the BF raceway. Two different lance designs and three different injection materials were studied as follows: conventional PC and two alternative carbon materials [15,16] using kinetic parameters deduced from thermal gravimetric analysis (TGA), and boundary conditions representing operational conditions used under BF trials. Model results were validated against high-speed camera measurements into the BF raceway, and the model indicated suitability to be used as part of a broader tool for evaluated PC and alternative carbon materials, as well as injection lances.

The 3D CFD model has been further used in comprehensive studies evaluating different PCs with varying volatile matter (VM) [17], and with injection of different types of biocoals [18,19]. In [18], PC mixed with biocoals were injected into the industrial BF of SSAB in Oxelösund with simultaneous temperature measurements using a thermovision camera (TVC), supplied by DIAS Infrared Systems, Dresden, Germany, focused into the raceway.

In previous studies, TVC was used to analyze raceway conditions such as the effect on PCR on temperatures [20] or to support CFD model development, but typically as separate efforts. While some have reconstructed temperature fields using CFD or thermal cameras, validation of raceway temperature from CFD model with TVC measurements has not been demonstrated. The inherent difficulty in pinpointing the exact TVC measurement location within the raceway further limits such validation approaches.

In the present work, a novel methodology is proposed to enable direct validation of the CFD model with TVC measurements. This approach aims to establish a reliable framework for future parametric studies of raceway behavior under varying operational conditions using CFD simulations. The focus is a multi-disciplinary approach, combining the use of both 3D CFD simulations of the raceway and experimental measurements in the form of TVC to be combined to cross-validate the results from both methods simultaneously.

## 2. Materials and Methods

### 2.1. Material and BF Process Variations

This study includes injection of a PC at SSAB BF No.4 in Oxelösund during chosen time periods, with simultaneous measurements into the raceway with a TVC. The chemical composition, in wt.%, of the PC was 80.6 C (C_fix_ 70.6), 4.1 H, 4.6 O, 2.2 N, volatile matter (VM) 18.5 and 8.2 Ash. The higher heating value of the PC was 32.3 MJ/kg. The PC was injected through swirl-tip oxy-coal lances, while a second dust lance was injecting nitrogen gas. Different process periods with varying raceway parameters were studied, and average values were deduced.

Four time periods, which correspond to a reference period, R1, a low oxygen to blast period, R2, a high blast moisture period, R3, and a higher PCI period, R4, were selected for modeling. As seen in Table 1, the PCI is around 121 g/Nm^3^ for the reference and other periods, except for R4, where the PCI is ~10 g/Nm^3^ higher. The blast flow, temperature, and oxygen to the lance are similar for all time periods, but the oxygen to the blast is lower for R2. The consumption of coke at the tuyere level is correspondingly lower when PCR increases, or oxygen enrichment decreases. The blast moisture is lowest in R2 and highest in R3. Table 2 shows BF parameters input into the Fluent CFD model.

### 2.2. Thermovision Camera

**Figure 1:**
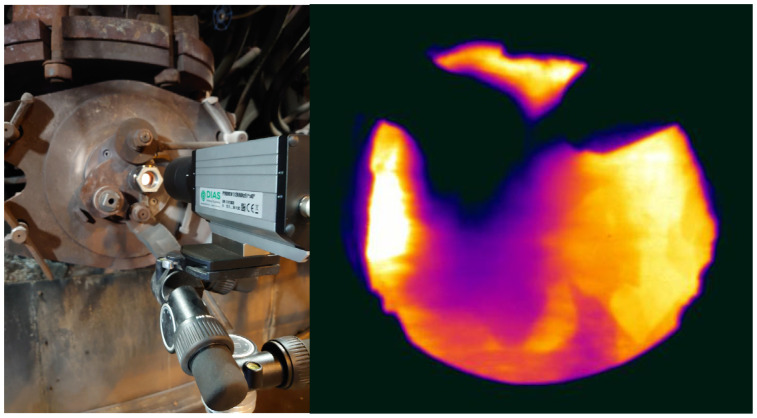
The thermovision camera, TVC, at the lid of the blowpipe looking into the raceway region through the tuyere (**left**). Example of thermal image obtained from TVC (**right**).

The validation of the CFD model is conducted by comparing images from a TVC positioned to measure through a viewing hole at the back of the blowpipe in the BF at SSAB Oxelösund, as shown on the left in Figure 1. Time-averaged temperature measurements from the TVC recorded over several hours are compared with corresponding locations from the CFD simulations. As an example, a snapshot of the thermal image obtained from TVC is shown on the right side of Figure 1.

The TVC used in this study, Model PYROVIEW 512N Compact+ is a 2D IR camera with a 512 × 384-pixel resolution, without the need for additional cooling, and capable of operating at two measurement ranges: 600–1500 °C or 1400–3000 °C, depending on the selected lens. Its spectral range is 0.8–1.1 um with a max frame rate of 60 Hz. A key advantage of this spectral range is that it allows the use of standard glass for the protective window, as these wavelengths are transmitted through regular glass. A specialized lens was employed to enable visualization of an area approximately 150 mm wide at a distance of 2 to 3 m, providing optimal view into the raceway. The tuyere diameter is approximately 150 mm.

The specified measurement cases, R1–R4, included long-time temperature measurement with the TVC. The measurement periods for validation of the CFD model were chosen to have as stable process and raceway parameter conditions as possible. The measured temperature is a short time average and the TVC settings are adjusted to focus on the similar position and depth for all monitored cases. Although operational settings and operational results are similar, in-furnace conditions vary over time due to factors such as shifting of the hot stove on blast, time relative to tapping, drifting in isothermal lines over time, and non-reduced material entering the lower part due to slips or scaffold peeling off. Also, the distribution of PC may vary due to the internal pressure distribution in front of tuyeres. This contributes to a fluctuating PC plume and may result in considerable variations in the measured temperature as well as the depth of the TVC measurement.

The measurement software PYROSOFT Professional (version 4.2.2.2) was used to define regions of interest (ROI) which can be points, lines, rectangles, circles/ellipsis, polygons, or free drawing. From ROI it is possible to define values of interest (VOI) which have been used in these measurements. For all VOI’s it is possible to have different information, for example, maximum, minimum, average, cross section temperatures, etc. With the software, it is possible to monitor and save temperature measurements. The sequence and formatting of file saving can be decided, such as sampling time, processing of temperature readings etc.

### 2.3. Methods

#### Model Setup

**Figure 2:**
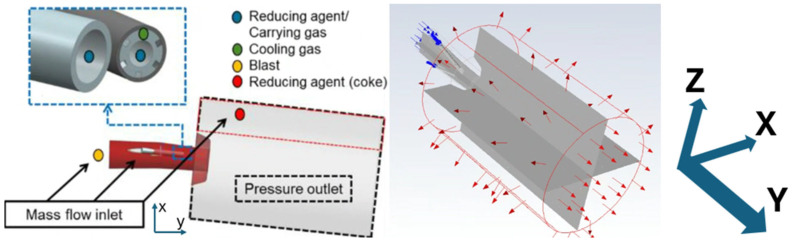
Computational domain, applied boundary conditions (red vector denotes outlet and blue vector denotes inlet), and contour plane with axis for the raceway-combustion model [17].

The multi-phase CFD simulations were performed using the commercial software ANSYS FLUENT version 18.2. A virtual geometry was constructed with simplified raceway with a cylindrical combustion chamber and open boundary, as seen in Figure 2. The continuous phase was modeled using 3D, steady-state Reynolds-averaged Navier–Stokes (RANS) equations with a realizable k-ε turbulence model and enhanced wall treatment. Alongside the RANS equations, energy and species transport equations were solved. Radiative heat transfer was modeled using the discrete ordinates radiation model.

The Euler–Lagrange approach was employed to simulate the multi-phase flow dynamics, treating the solid phase in a steady-state manner by tracking spherical particles across the computational domain. These particles interacted with the continuous phase through mass, momentum, and energy exchange. A stochastic tracking model accounted for turbulence-induced dispersion, while a particle radiation interaction model incorporated particle effects into the radiation model. To accurately reflect the particle size distribution (PSD), a Rosin–Rammler distribution was applied based on PSD data from material screening. The CFD model employed in this study has been previously developed and described in detail in [15,16,17,18].

The conversion of pulverized reduction agents is defined by four steps in the model: pre-heating, devolatilization, homogenous reactions, and heterogeneous reactions. The chemical reactions considered are presented in Table 3.

Kinetic parameters for reaction (CR1), (CR7), and (CR8) were deduced from TGA data on sample mass versus time in different atmospheres, as shown in Table 4. Coke has minor volatile content thus the devolatilization is not considered in the model. Previous publications provide a comprehensive explanation of the governing equations, boundary conditions, and reaction mechanisms used in the model [16,17,18,19]. Readers seeking a deeper understanding of the model formulation and underlying assumptions are encouraged to refer to these sources.

## 3. Results and Discussion

### 3.1. Raceway Size

The performance of the different simulation cases is investigated through determining the raceway size and comparing it between the cases. In these cases, the raceway size is defined as the region within the raceway part of the BF where the oxygen content is above 0.5%. There is thus no consideration on the temperature or other gas components.

**Figure 3:**
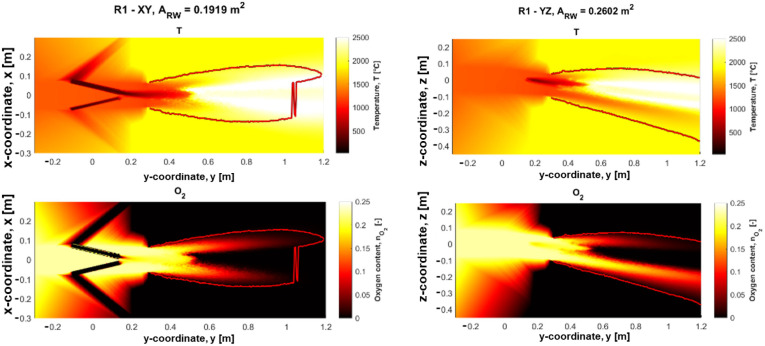
Temperature and gas composition fields for the XY- and YZ-planes of the raceway of reference case R1.

**Figure A1:**
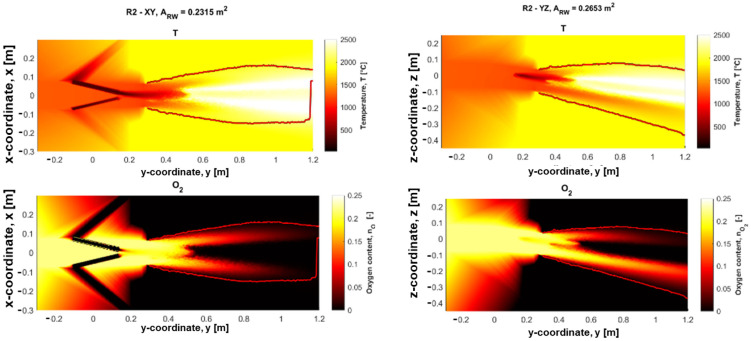
Temperature and gas composition fields for the XY- and YZ-planes of the raceway of case R2, low oxygen.

**Figure A2:**
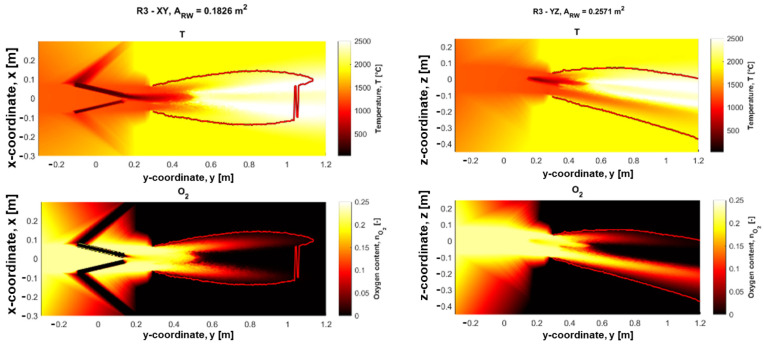
Temperature and gas composition fields for the XY- and YZ-planes of the raceway of case R3, high blast moisture.

**Figure A3:**
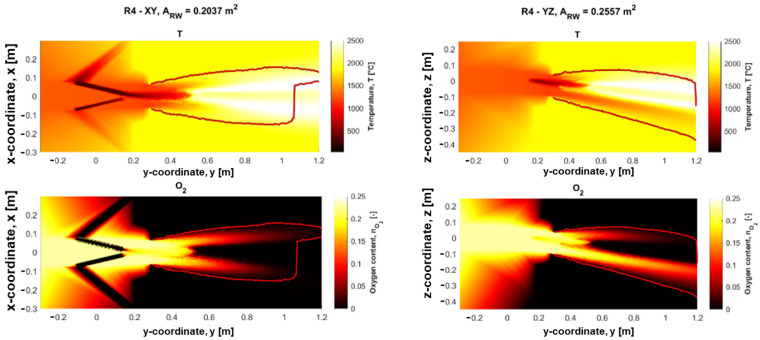
Temperature and gas composition fields for the XY- and YZ-planes of the raceway of case R4, high PCI no steam.

The temperature and gas composition fields for CFD simulation of the reference case R1 are presented in Figure 3 for the XY (the horizontal plane through the lances, as seen from above) and YZ (the vertical plane through the center of the tuyere, as seen from the side) planes, respectively. The temperature and gas compositions for R2–R4 are illustrated in Figure A1, Figure A2 and Figure A3 (Appendix A). The raceway boundaries are represented by the red lines and ARW denotes the area of the bounded raceway region.

The selected parameters describing the size of the raceway are the length of the upper and lower oxygen jets as well as the distance to the start of the central oxygen deficient corridor and the raceway area in both the XY- and YZ-planes. The respective values of these parameters for the four different cases are presented in Table 5.

It is indicated by the model that lowering the oxygen in case R2, which also results in a corresponding reduction in the consumption of coke per time unit, see Table 2, results in an overall larger raceway region, as seen mainly in the XY plane. This is likely related to less favorable conditions for combustion due to lower oxygen content, which displaces the start of combustion further into the raceway, and overall, slightly reduces the temperatures in the raceway. Kinetic parameters that are characteristic of a lowly reactive coke result in lower consumption of oxygen per time unit and thus the raceway extends deeper into the BF.

The higher moisture addition to the blast in R3 contributed to reduced raceway size in both planes. This is expected behavior, as more moisture results in a higher energy requirement to supply the latent heat to increase the additional water’s temperature, evaporate it, and dissociate it, forming oxygen and hydrogen.

The coke consumption is decreased in direct correlation to increasing the PCR in R4. The oxygen deficient corridor started closer to the tuyere nose than for the reference in one plane, but later in the other plane. An overall conclusion that can be drawn is that the PC is combusted more rapidly in this case.

### 3.2. Temperature Validation with Thermovision Camera Data

**Figure 4:**
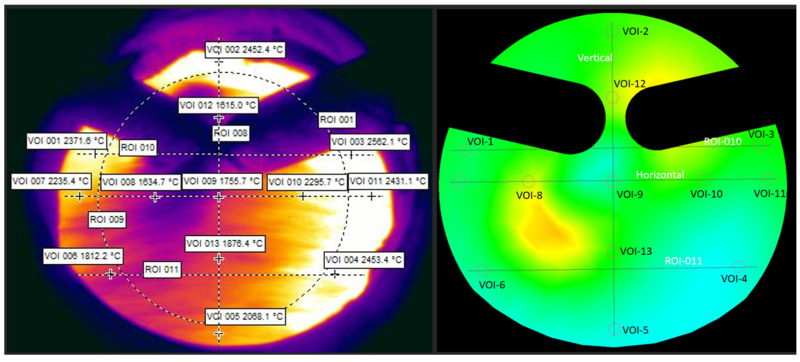
Example of images used for validation of the CFD model, a snapshot from thermovision camera (**left**) and the same plane extracted from CFD model (**right**).

Temperatures at each of 13 VOIs were extracted from the TVC and are shown in the snapshot to the left in Figure 4. The data corresponding to a similar plane extracted from the CFD model is pictured to the right in Figure 4.

A comparison between the temperature data, VOI, of the TVC to the CFD model is shown in Table 6, whereas the CFD results are extracted from the simulations on a plane 0.35 m from the tip of the lance. From the overall trends, the TVC measurements indicated higher temperatures for most measurements. The exceptions are VOI 2, which lies on the top edge of the tuyere, as well as VOI 8, 12, and 13, which all lie in the region affected by the plume. This indicates that the TVC is restricted from measuring deeper into the center of the raceway when there is a coal plume present. The resulting temperature from the points in the plume may however still be useful to determine the temperature at the plume in the raceway.

The variation between the different periods as well as the overall magnitude of the temperature is larger in the TVC measurement compared to the steady-state results of the CFD model. The data from the points shows that the variation between the TVC measurements is in the region of 350–500 °C compared to for the CFD simulation where the variation is generally within 100 °C. The steady state CFD model result shows the variation due to the operational settings, while the TVC also captures instantaneous temperature changes in raceway due to in-furnace phenomena influencing the pressure and therefore the PCI and blast are difficult to simulate.

The measurement uncertainty of the thermal camera is approximately ±2% of the recorded temperature in Celsius, which corresponds to an uncertainty range of ±40–50 °C for the temperature values encountered in this study. The CFD simulation relative error was calculated as the absolute difference between the CFD-model temperature and the TVC temperature, divided by the TVC temperature. The average relative error values range from 5% to 25%. These differences may be attributed to factors such as the exact location of the analysis point, variations in the plume, and other process parameters not included in the model.

**Figure 5:**
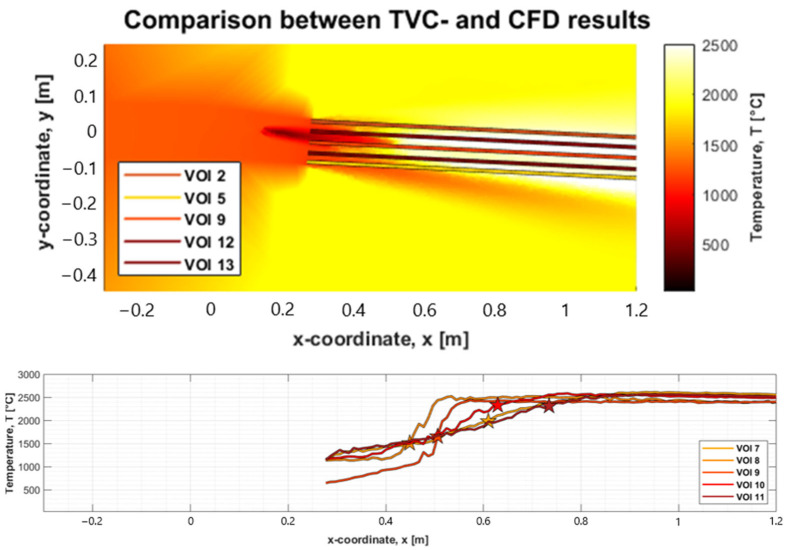
Above, lines on the YZ-plane used for extraction of temperature data for validation of the model. Below, identification of depth of TVC measurement using the temperature graphs from simulation indicated by the stars which correlate to the temperature registered in the experiment.

The TVC is set up to measure into the raceway in similar ways during all test periods R1–R4; however, the plume may block the camera’s vision at one VOI, making it measure shallowly within the tuyere whilst at another point it measures unobstructed deeper into the BF. As a result, the measuring depth of the TVC may vary between selected process periods. To investigate the effect of this, it is instead necessary to extract the comparative values from the CFD by extracting the temperature data from lines penetrating parallelly into the raceway, rather than points on a plane with fixed distance from the tuyere, which may or may not be representative. In this case the lines were created with the same angle as the tuyere exit, and they do not take into consideration the expanding field of vision of the camera. The constraint of the lines is that they pass through the x- and y-coordinates of respective previously compared points (VOI 1–VOI 13) at the tuyere exit. Figure 5 shows the lines which are used for temperature extraction, and identification of depth of TVC measurement using the temperature graphs from simulation indicated by the stars which correlate to the temperature registered in the experiment.

**Figure 6:**
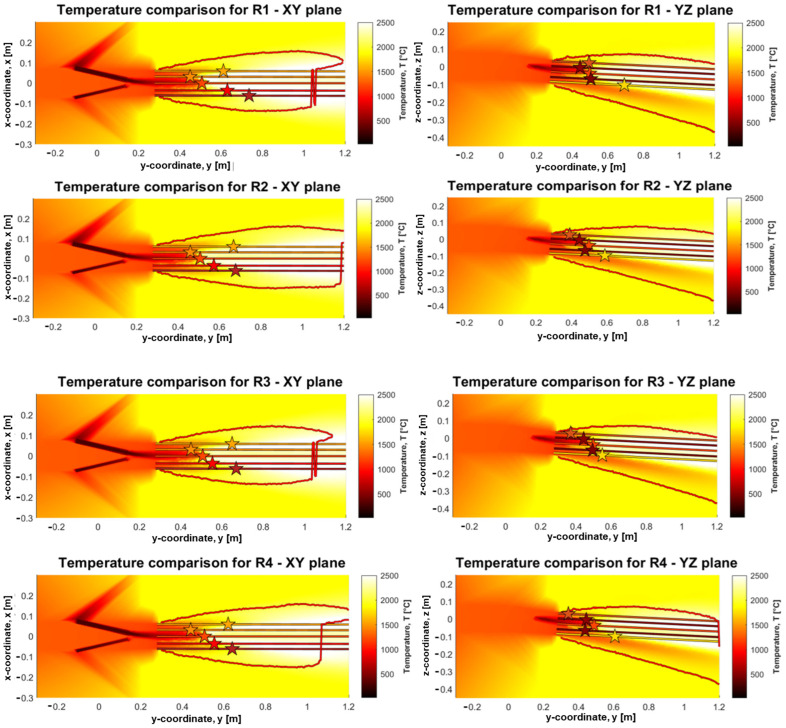
CFD temperature lines from the XY- and YZ-planes compared with average temperature at the TVC measurement points, the stars indicate where the CFD model temperature matches the measured TVC average temperature. From top to bottom R1, R2, R3 and R4.

Comparing the temperature measurements from the TVC with the CFD, the temperatures are plotted along the lines shown in both the XY- and YZ-planes. The average temperature is then extracted from the TVC in each of the compared points (VOI 1–VOI 13), as performed previously, and matched to the first point on the projected line from the CFD results with the similar temperature. The related intersection point is marked by a star on each line, see Figure 6. This gives an indication of the depth of the measurement for the XY- and YZ-planes, respectively, which can be used to determine if the depth is reasonable as depth of TVC measurement.

The respective distances are additionally presented in Table 7 for R1 (reference), R2 (low O_2_), R3 (high moisture), and R4 (high PC), with their respective simulation equivalent.

While it is not possible to exactly determine a fixed depth within the raceway where the TVC is indicated, nor is it possible to completely validate the CFD model results against the camera measurement, it is possible to obtain some indications on the measurement based on the results.

The combination of simulated and experimental results indicate that the TVC is capable of measuring at a distance ranging between 0.5 and 0.7 m into the raceway, where the zero-depth reference point is located at the tip of the lance. The coal plume blocks the camera’s view in the central regions of the raceway, preventing deeper measurements into the raceway. The restriction in measuring depth was more at higher PC rates. The YZ-plane graphs suggest that there is a nearly linear trend towards increased measurement depth in the downwards vertical direction of the raceway. The XY-plane graphs similarly show a linear trend, whereas the measurement depth increases from the left towards the right of the raceway, relative to an outside observer. This is with the exception of VOI 7, which is the leftmost point, and likely in a zone that is outside the coal plume. Overall, this indicates that the coal plume occupies the upper-central left region of the raceway, relative to an observer looking into the raceway from outside.

The TVC data indicates that the temperature within the raceway is the highest for the reference period, R1. The temperature is significantly lower for all other periods, with an average temperature decrease of 163, 181, and 210 °C, for R2, R3, and R4, respectively. This is in line with the fact that lowering the O_2_ concentration, increasing the moisture addition, and increasing PCR all contribute to lower raceway adiabatic temperature (RAFT). However, the effect on measured temperature is higher compared to an estimate based on rule of thumb which is <100 °C [2]. The higher effect seen in the measured data can be explained that the RAFT corresponds to an average for the raceway, while the impact is likely strongest close to the tuyere nose due to evaporation and cracking of moisture and devolatilization of PC.

Overall, it is indicated that the behavior of the plume is important to consider when evaluating temperature measurements into the raceway to be able to quantify the dynamics of the raceway region of the BF and verify simulation data for raceway.

## 4. Conclusions

The combined use of TVC and CFD simulations was applied to gain insight into the dynamics of the raceway region and to further verify raceway modeling results. These indicate that it is possible to measure the temperature within the raceway region, using TVC at depths indicated to be 0.5–0.7 m when not obscured by the coal plume. An increase in PCR is seen to have clear effects on the data achieved from TVC, due to the focus of the measurement ending up on the plume. The measurements are clearly quantitatively affected by lower temperatures when measuring at the coal plume compared to deeper in the raceway.

The temperature differences in raceway average temperatures are in the order of 200 °C between similar process conditions. This can be compared to expected differences based on modeling results and conventional RAFT calculation in the region of <100 °C for the changes conducted. However, the impact on temperature is stronger in a position close to the tuyere nose compared to on the average of the whole region.

The conclusions regarding the impact on raceway size assume that the raceway boundary corresponds to the border at which the remaining O_2_ content is less than 0.5%. Furthermore, the extension of the raceway is judged for the X-Y- and Y-Z-planes, respectively, not on a continuous 3D surface of the raceway. The simulations indicate that the decrease of oxygen enrichment to the blast results in an extended raceway region, which can be due to less chemically favorable conditions for combustion, offsetting the ignition point and reducing the reaction rate in the raceway. An increased moisture content in the blast results in a reduced size of the raceway region as more energy is consumed as latent energy and to crack the water. Finally, the increase in PCR results in an inconclusive difference in raceway size in evaluated planes.

Overall, it could be seen that the raceway region is difficult to quantify even with a combined simulative and experimental effort. However, the synergy of using this approach is obvious, allowing for a more in-depth understanding of where the measurements take place via cross-validation. Future investigations into the raceway dynamics can thus clearly benefit from utilizing this multi-disciplinary approach. Further evaluation of the impact of the coal plume and its interaction with the TVC results would be beneficial.

## Figures and Tables

**Table 1:** Pulverized coal injection rate (PCR), and raceway parameters on average during chosen time periods.

Parameter [Unit]	R1 (Reference)	R2 (Low Oxygen to Blast)	R3 (High Blast Moisture)	R4 (High PCI)
Pulverized Coal Injection Rate, PCR [g/Nm^3^]	121.6	120.8	121.6	130.5
Blast flow [kNm^3^/h]	119.6	119.6	119.7	119.6
Blast moisture [g/Nm^3^]	6.61	4.2	14.1	6.8
O_2_ to blast [kNm^3^/h]	2.87	1.75	2.88	2.88
O_2_ to lance [kNm^3^/h]	2.379	2.380	2.379	2.379

**Table 2:** Parameters inserted into the CFD model corresponding to the average BF parameters for each time-period, varied parameters are in bold italics.

Variable	R1 (Reference)	R2 (Low Oxygen to Blast)	R3 (High Blast Moisture)	R4 (High PC, No Steam)
$\mathrm{PCI}/{\dot{m}}_{PC}$ [g/s]	202.2	200.7	202.1	***216.9***
${\dot{m}}_{coke}$ [g/s]	1633.0	1245.9	1633.6	1619.7
${\dot{m}}_{O2,lance}$ [g/s]	42.5	42.6	42.6	42.6
${\dot{m}}_{blast}$ [g/s]	2138.6	2139.5	2132.0	2139.4
Blast water content [%]	0.83%	0.53%	***1.75%***	***0.85%***
Blast oxygen content [%]	22.73%	***22.05%***	22.53%	22.72%
Blast nitrogen content [%]	76.44%	77.42%	75.72%	76.43%
Blast temperature [°C]	1027.1	1027.9	1026.5	1027.0

**Table 3:** Chemical reactions taking place in the CFD model [16,17,18,19].

**Devolatization**	
${Pulverized\;coal\;\;}_{\to \;Char\;(C_{\left(s\right)})\;+\;Residue\;(Ash)\;}^{\to \;Volatile\;Matter\;(VM)}$	(CR1)
**Homogenous reactions**	
$VM_{PC}+\;\alpha O_{2}\to \beta CO+\zeta CO_{2}+\delta \;H_{2}O+\varepsilon N_{2}+\eta SO_{2}$	(CR2)
$CO+0.5\;O_{2}\to CO_{2}$	(CR3)
$H_{2}+0.5\;O_{2}\to H_{2}O$	(CR4)
$CO+H_{2}O\to CO_{2}+H_{2}$	(CR5)
$CO_{2}+H_{2}\to CO+H_{2}O$	(CR6)
**Heterogeneous reactions**	
$C_{\left(s\right)}+O_{2}\to CO_{2}$	(CR7)
$C_{\left(s\right)}+CO_{2}\to 2CO$	(CR8)
$C_{\left(s\right)}+H_{2}O\to CO+H_{2}$	(CR9)

**Table 4:** Kinetic parameters for heterogeneous reactions deduced from TGA test results [19].

Reaction	Devolatilization				Combustion			Gasification		
Parameter	Start of Reaction [°C]	Exp. Factor [1/s]	Norm. Exp. Factor [1/s]	Activation Energy [kJ/mol]	Exp. Factor [s/m]	Norm. Exp. Factor [s/m]	Activation Energy [kJ/mol]	Exp. Factor [s/m]	Norm. Exp. Factor [s/m]	Activation Energy [kJ/mol]
PC	340	1.80 × 10^3^	1.96 × 10^3^	89	5.55 × 10^−3^	2.18 × 10^3^	109	1.10 × 10^−3^	7.62	170
Coke	-	-	-	-	234	234	90	11	11	240

**Table 5:** Resulting raceway comparison from the R1–R4 simulations.

	Parameter	R1—Reference	R2—Low O_2_	R3—High H_2_O	R4—High PCI
**XY-plane**	Upper O_2_ jet depth [m]	1.19	Out of bounds	1.15	Out of bounds
	Lower O_2_ jet depth [m]	1.04	1.18	1.03	1.06
	Central O_2_ deficient corridor start depth [m]	0.55	0.55	0.56	0.53
	Raceway area [m^2^]	0.192	0.232	0.183	0.204
**YZ-plane**	Upper O_2_ jet depth [m]	Out of bounds	Out of bounds	Out of bounds	Out of bounds
	Lower O_2_ jet depth [m]	Out of bounds	Out of bounds	Out of bounds	Out of bounds
	Central O_2_ deficient corridor start depth [m]	0.52	0.55	0.54	0.57
	Raceway area [m^2^]	0.261	0.265	0.257	0.256

**Table 6:** Average temperatures for the VOI from the TVC and CFD model along with relative error.

Point	TVC Temperature [°C]				CFD Model Temperature [°C]				CFD Relative Error [%]			
	R1	R2	R3	R4	R1	R2	R3	R4	R1	R2	R3	R4
VOI 2	1739	1439	1432	1389	1718	1664	1753	1744	−1%	16%	22%	26%
VOI 5	2027	1578	1425	1692	1308	1301	1290	1323	−35%	−18%	−9%	−22%
VOI 7	1982	2092	2141	1993	1516	1451	1502	1500	−24%	−31%	−30%	−25%
VOI 8	1503	1444	1463	1393	1856	1884	1894	1709	23%	30%	29%	23%
VOI 9	1714	1680	1661	1589	1571	1440	1433	1367	−8%	−14%	−14%	−14%
VOI 10	2325	2070	2014	1973	1571	1588	1627	1573	−32%	−23%	−19%	−20%
VOI 11	2334	2089	2100	2020	1542	1499	1559	1553	−34%	−28%	−26%	−23%
VOI 12	1694	1541	1556	1609	1858	1848	1876	1796	10%	20%	21%	12%
VOI 13	1824	1750	1723	1608	1714	1713	1707	1740	−6%	−2%	−1%	8%
Average	1905	1742	1724	1696	1628	1599	1289	1589	−15%	−8%	−25%	−6%

**Table 7:** Depth relative injection-lance tip at which the CFD model estimated temperature matches the TVC average temperature.

Point	Corresponding Depth in CFD [m]			
	R1	R2	R3	R4
VOI 2	0.493	0.388	0.369	0.340
VOI 5	0.691	0.586	0.548	0.436
VOI 7	0.610	0.667	0.648	0.620
VOI 8	0.449	0.458	0.449	0.440
VOI 9	0.506	0.572	0.506	0.506
VOI 10	0.629	0.506	0.553	0.553
VOI 11	0.735	0.677	0.668	0.639
VOI 12	0.443	0.443	0.443	0.443
VOI 13	0.503	0.474	0.493	0.436
Average	0.562	0.530	0.520	0.490

## Data Availability

The datasets presented in this article are not readily available due to sensitivity in sharing geometrical designs and process conditions.

## Abbreviations

The following abbreviations are used in this manuscript:

3D	Three-dimensional
BF	Blast furnace
CFD	Computational fluid dynamics
PC	Pulverized coal
PCI	Pulverized coal injection
PCR	Pulverized coal injection rate
PSD	Particle size distribution
RANS	Reynolds-averaged Navier–Stokes
ROI	Region of interest
TVC	Thermovision camera
VOI	Value of interest
VM	Volatile matter
